# A Case of T/NK-Cell Post-Transplantation Lymphoproliferative Disease 7 Years after Heart Transplantation

**DOI:** 10.3390/jcdd9020038

**Published:** 2022-01-24

**Authors:** Makiko Nakamura, Teruhiko Imamura, Kohji Takagi, Masakazu Hori, Shinichi Tanaka, Joji Imura, Koichiro Kinugawa

**Affiliations:** 1 The Second Department of Internal Medicine, University of Toyama, 2630 Sugitani, Toyama 930-0194, Japan; nakamura@med.u-toyama.ac.jp (M.N.); masahori6059@yahoo.co.jp (M.H.); kinugawa@med.u-toyama.ac.jp (K.K.); 2 Department of Diagnostic Pathology, University of Toyama, 2630 Sugitani, Toyama 930-0194, Japan; Ktakagi@med.u-toyama.ac.jp (K.T.); shinichi@med.u-toyama.ac.jp (S.T.); imura@med.u-toyama.ac.jp (J.I.)

**Keywords:** Epstein–Barr virus, cardiomyopathy, immunosupressant, hemophagocytosis

## Abstract

Post-transplant lymphoproliferative diseases (PTLD) are potentially fatal complications after cardiac transplantation. Most cases are Epstein–Barr virus (EBV)-related B-cell tumors, and reduction of immunosuppression treatment as well as the use of rituximab in combination with other chemotherapy are effective. However, patients with T/NK-cell PTLD post-cardiac transplantation are rarely reported. We had a patient with a fever that lasted for three weeks, with lung infiltrations and hepatosplenomegaly, who had EBV-associated hemophagocytosis 7 years after heart transplantation and was eventually diagnosed with T/NK-cell PTLD by autopsy. Although rare diseases, regular monitoring of EBV-DNA levels might be crucial for early diagnosis and treatment of PTLD.

## 1. Introduction

Post-transplant lymphoproliferative diseases (PTLD) are heterogeneous lymphoid disorders ranging from polyclonal proliferations to aggressive lymphomas, secondarily developing following solid organ/hematopoietic transplantation [1]. Most PTLD are Epstein–Barr virus (EBV)-related B-cell tumors, dominantly triggered by immunosuppressive therapy. PTLD can be fatal complications, particularly for those with EBV-seronegative at baseline, such as pediatric candidates [1,2]. 

Timely and accurate diagnosis based on histological examination of biopsy tissue, including immunostaining of EBV and evaluation for clonality, is essential for early intervention [2]. A reduction in immunosuppression remains a mainstay. Since PTLD is usually due to neoplastic proliferation of B-cells, rituximab-incorporated chemotherapy is also recommended [1,2].

A few PTLDs (7–15% of all PTLDs following solid organ transplantation) have their origins in T/NK-cells instead of B-cells. Most T/NK-cell PTLDs (69%) were post-renal transplantation and 20% were following heart or heart–lung transplantations [3]. These atypical PTLDs occur later than the typical B-cell PTLD. Survival in patients with these atypical PTLDs is worse than that of the typical B-cell PTLD [3,4]. However, little is known about the T/NK-cell PTLDs following heart transplantation. We encountered a patient who was EBV-seronegative at baseline and developed T/NK-cell PTLD, which was diagnosed by the autopsy 7 years following heart transplantation.

## 2. Case Report

### 2.1. Heart Transplantation (7 Years Ago)

A 28-year-old man who had refractory heart failure due to doxorubicin-induced cardiomyopathy received heart transplantation. He was seronegative for EBV at transplantation and 6 months later, after which EBV antibody titer level was not measured. He received a standard immunosuppressant regimen including cyclosporine, everolimus, and prednisolone. 

### 2.2. Adjustment of Immunosuppression Treatment

Five years ago, cyclosporine was changed to tacrolimus. Three years ago, steroid pulse therapy was performed to treat acute cellular rejection with ISHLT grade 3A. Following the steroid pulse therapy, he did not have a significant rejection reaction under the following immunosuppressant regimen: tacrolimus trough 4–6 ng/mL, everolimus trough 5–7 ng/mL, and prednisolone of 5 mg/day. 

### 2.3. Fever and Diagnosis of Pneumonia (1 Month Ago)

**Figure 1:**
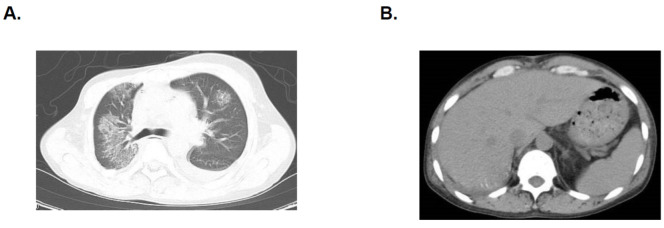
Computed tomography imaging before referral. Infiltration shadows on bilateral lung with pleural effusion (**A**) and hepatosplenomegaly (**B**) were observed.

He complained of high fever and dry cough for over 2 weeks refractory to multiple antibiotics. Chest computed tomography obtained in the former institute showed progressive bilateral lung infiltration and pleural effusion accompanying hepatosplenomegaly (Figure 1A,B). He was referred to our institute for further treatment.

### 2.4. On Admission

Oxygen saturation was 98% (nasal oxygen 2 L/min) and superficial lymph nodes were not palpable. The laboratory data showed pancytopenia and hepatorenal dysfunction. Serum ferritin was 3036 ng/mL, triglyceride was 363 mg/dL, and d-dimer was 8.5 μg/mL. The EBV DNA level in peripheral blood was 1.6 × 10^6^ copy/mL, whereas cytomegalovirus antigen was negative. EBV capsid antigen-IgM, EBV capsid antigen-IgG, and EBV nuclear antigen (EBNA) were <10-hold, 640-hold, and <10-hold, respectively.

### 2.5. Diagnosis of EBV-Associated Hemophagocytic Syndrome

**Figure 2:**
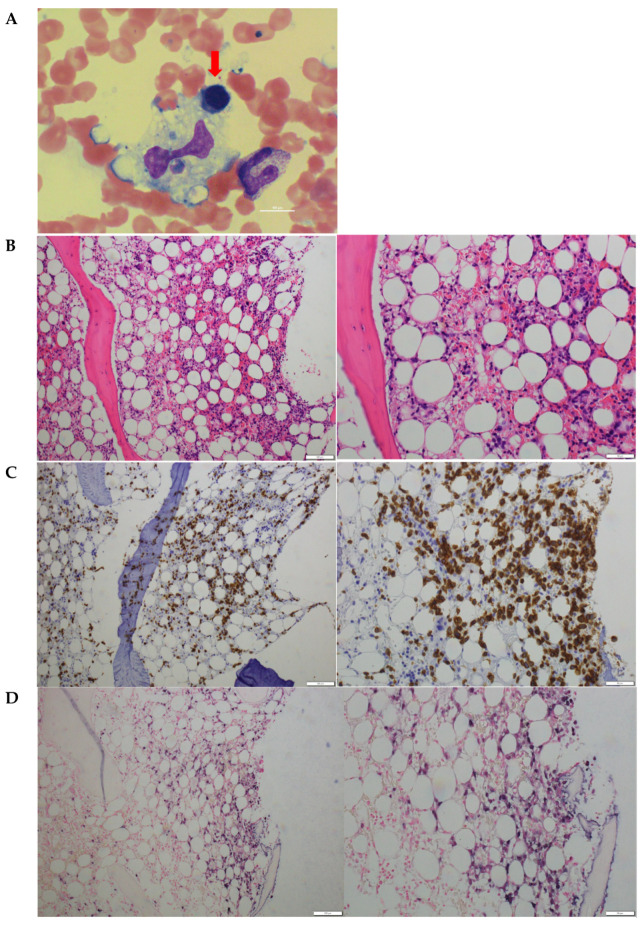
Hemophagocytosis was found in bone marrow biopsy smear (May-Giemsa staining, (**A**)). Lympho-proliferation without dysplasia with CD3 and EBV positive cells in bone marrow biopsy (Hematoxylin-eosin staining, (**B**); CD3 immunostaining, (**C**); EBER-1 in situ hybridization, (**D**)) were shown.

A bone marrow test was performed to investigate his pancytopenia and he was diagnosed with hemophagocytic syndrome (Figure 2A). There was significant proliferation of CD3-positive T cells, which did not align with dysplasia (Figure 2B,C). There was no proliferation of CD20-positive B cells. EBER-1 was positive in situ hybridization (Figure 2D). He was diagnosed with EBV-associated hemophagocytic syndrome.

The trough levels of tacrolimus and everolimus were elevated to 14.6 ng/mL and 16.9 ng/mL, respectively, and they were temporarily discontinued. Steroid pulse therapy consisting of methylprednisolone 1 g/day for 3 days was conducted, followed by oral prednisolone (60 mg/day). Intravenous immunoglobulin and granulocyte-colony stimulating factors were administered. 

Despite multidisciplinary therapy, his dyspnea symptom progressed accompanying multi-organ failure and disseminated intravascular coagulation, which were refractory to mechanical ventilation and continuous hemodiafiltration. He eventually passed away on day 8.

### 2.6. Diagnosis of T/NK-Cell PTLD on Autopsy

**Figure 3:**
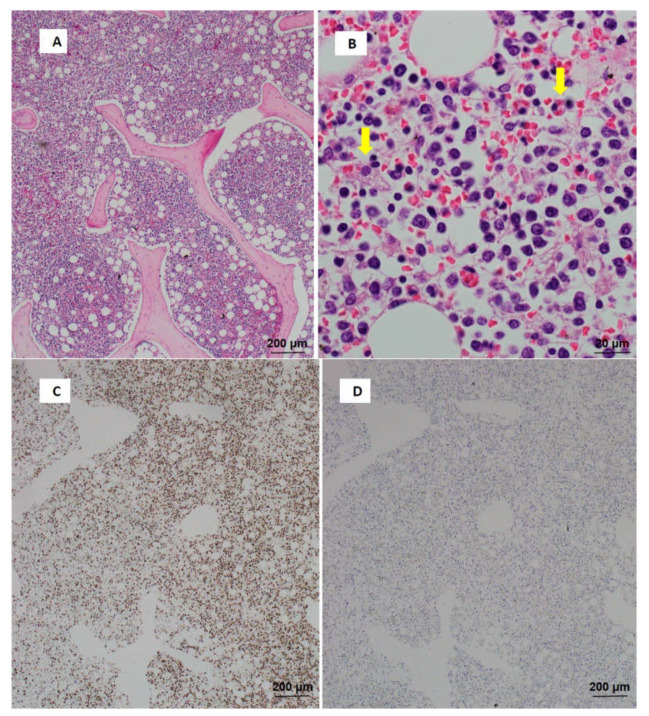

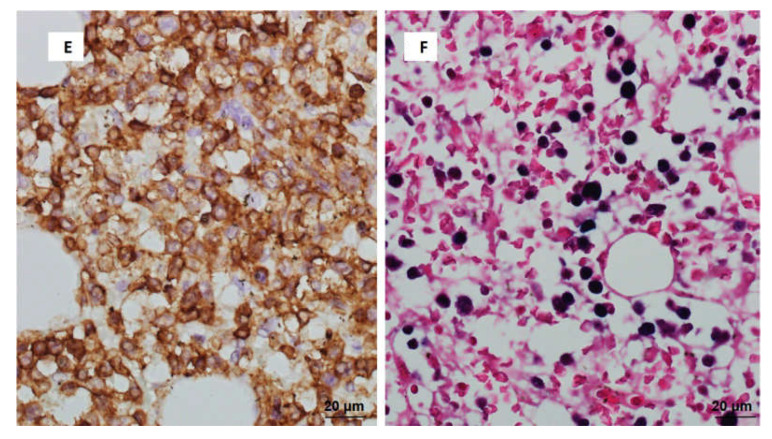
There were small to medium atypical lymphocyte aggregation in bone marrow (**A**). Diffuse lymphocytic infiltration with hemophagocytosis (arrow sign) was also scattered (**B**). Atypical lymphocytes were positive for CD3 (**C**) and negative for CD20 (**D**). There were also positive for CD8 (**E**) and EBER in situ hybridization (**F**).

The autopsy showed hemophagocytosis in the bone marrow, liver, spleen, and lung. Also observed was small to medium atypical lymphocyte aggregation with CD3, CD8, and EBER-in situ hybridization-positive. CD5 and CD20-negative were found in bone marrow, heart, liver, spleen, kidney, and lung (Figure 3A–F). He was finally diagnosed with monomorphic PTLD with T/NK-cell and diffuse alveolar damage.

## 3. Discussion

### 3.1. T/NK-Cell PTLD

In the 2016 revision of the World Health Organization Classification of lymphoid neoplasms, PTLDs are classified into six subcategories: (1) plasmacytic hyperplasia; (2) infectious mononucleosis; (3) florid follicular hyperplasia; (4) polymorphic; (5) monomorphic including B- and T-/NK-cell types; and (6) classical Hodgkin lymphoma [5]. 

In our patient, monomorphic CD3-positive T lymphocyte aggregation was observed and he was diagnosed with monomorphic T-/NK-cell type PTLD. This type of PTLD is quite rare constituting between 7% and 15% of all PTLD, dominantly following kidney transplantation [3].

### 3.2. EBV Infections

EBV is one of the major viral triggers of PTLD. The host of EBV is generally a B-cell, whereas some T/NK-cell PTLDs are also triggered by the EBV infection [3,6]. In our patient, he was uninfected by EBV until 6 months after heart transplantation. On admission to our hospital, he was positive for virus capsid antigen-IgG, instead of IgM, whereas EBNA was negative, with a higher EBV DNA level. He may have been infected with EBV for the first time after 6 months post-transplant but may not be able to produce EBNA due to immunosuppression. Or he was at a recovery phase of the primary EBV infection at the time of index admission. 

### 3.3. EBV-Associated Hemophagocytic Syndrome and PTLD

His clinical and histopathological presentation were hemophagocytic syndrome, which is a fulminant life-threatening inflammatory disease due to uncontrolled hyperactivation of the immune system. Its diagnosis is based on the presence of the following criteria: fever, splenomegaly, cytopenias, hepertriglycemia or hyperfibrinogenemia, hemophagocytosis, low or absent NK-cell activity, hyperferritinemia, and soluble CD 25 levels [6]. 

EBV-associated hemophagocytic syndrome is rare and mainly affects children and adolescents in Asia. EBV-infected cells are small and slight or no atypia, and can be observed in many organs. These cells often have a cytotoxic CD8-positive T-cell origin [6]. Some patients’ conditions evolve other EBV-positive lymphoproliferative diseases requiring more intensive therapies, and systemic EBV-positive T/NK-cell lymphoma of childhood may also occur at the same time as EBV-hemophagocytic syndrome, whereas survival for patients with EBV-associated hemophagocytic syndrome will improve when treated promptly following the HLH-2004 protocol [6,7]. 

In our patient, CD8-positive cytotoxic T-cells in addition to CD3-positive T-cells were also detected in bone marrow and other organs by autopsy, although T-cells with CD3 found by bone marrow test were not apparently dysplastic. He might have had primary EBV infection shortly before he had a fever and developed hemophagocytic syndrome.

### 3.4. Detection and Treatment of T/NK-Cell PTLD

Clinical presentation and response to treatment of T/NK-cell PTLD vary in each case [3]. It is sometimes challenging to diagnose with T/NK-cell PTLD when the patient is alive, as with our patient, but scheduled monitoring of EBV antibody titer and DNA levels as well as systemic computed tomography imaging to survey lymphoid proliferation might be useful for early detection of T/NK-cell PTLD, especially in patients with EBV-seronegative at transplantation. 

The quantification of circulating EBV DNA loads has played an important role in the diagnosis and management of EBV-associated PTLD. However, there is a lack of standardization, and the optimal specimens for measuring viral loads are unknown. The newly established World Health Organization standard for EBV quantification will encourage collaborative studies across institutions and countries to establish proper guidelines for EBV diagnosis and treatment [8].

Further studies are warranted to construct optimal surveillance and therapeutic strategy for T/NK-cell PTLD following heart transplantation.

## 4. Conclusions

PTLD is a potentially fatal complication after cardiac transplantation. Of note, we had a patient with EBV-related T/NK-cell monomorphic PTLD accompanying hemophagocytic syndrome. Scheduled monitoring of EBV DNA levels might be useful for early detection and diagnosis of PTLD. 

## Data Availability

Data are contained within the article.
